# Exploring paramedic perceptions of feedback using a phenomenological approach

**DOI:** 10.29045/14784726.2020.06.5.1.7

**Published:** 2020-06-01

**Authors:** Peter Eaton-Williams, Freda Mold, Carin Magnusson

**Affiliations:** South East Coast Ambulance Service NHS Foundation Trust: ORCID iD: https://orcid.org/0000-0001-5664-3329; University of Surrey: ORCID iD: https://orcid.org/0000-0002-6279-5537; University of Surrey

**Keywords:** continuing professional development, feedback, paramedic

## Abstract

**Objectives:**

Despite widespread advocacy of a feedback culture in healthcare, paramedics receive little feedback on their clinical performance. Provision of ‘outcome feedback’, or information concerning health-related patient outcomes following incidents that paramedics have attended, is proposed, to provide paramedics with a means of assessing and developing their diagnostic and decision-making skills. To inform the design of feedback mechanisms, this study aimed to explore the perceptions of paramedics concerning current feedback provision and to discover their attitudes towards formal provision of patient outcome feedback.

**Methods:**

Convenience sampling from a single ambulance station in the United Kingdom (UK) resulted in eight paramedics participating in semi-structured interviews. Interpretative phenomenological analysis was employed to generate descriptive and interpretative themes related to both current and potential feedback provision.

**Results:**

The perception that only exceptional incidents initiate feedback, and that often the required depth of information supplied is lacking, resulted in some participants describing an isolation of their daily practice. Barriers and limitations of the informal processes currently employed to access feedback were also highlighted. Formal provision of outcome feedback was anticipated by participants to benefit the integration and progression of the paramedic profession as a whole, in addition to facilitating the continued development and well-being of the individual clinician. Participants anticipated feedback to be delivered electronically to minimise resource demands, with delivery initiated by the individual clinician. However, a level of support or supervision may also be required to minimise the potential for harmful consequences.

**Conclusions:**

Establishing a just feedback culture within paramedic practice may reduce a perceived isolation of clinical practice, enabling both individual development and progression of the profession. Carefully designed formal outcome feedback mechanisms should be initiated and subsequently evaluated to establish resultant benefits and costs.

## Introduction

Paramedics are rarely provided with feedback on the accuracy of their diagnoses or the consequences of their decision-making (Cash et al., 2017; O’Hara et al., 2015). Yet feedback on an individual’s performance has been advocated as fundamental to the delivery of high quality healthcare, as it facilitates reflection on practice and learning (Flottorp et al., 2010). Feedback may also enhance levels of work engagement and well-being (Bakker et al., 2014; King’s Fund, 2015).

Many paramedics express a desire to learn more information about the outcomes of incidents after their involvement has ended, for both emotional and developmental motives (Morrison et al., 2017; O’Hara et al., 2015). This ‘outcome feedback’ is most commonly gained through the use of informal processes that have limitations to their effectiveness and potential barriers to gaining provision (Ivers et al., 2012; Morrison et al., 2017). However, a small number of formal mechanisms providing outcome feedback from emergency departments (EDs) have been established in the United Kingdom (UK) (Pollard & Black, 2015; Princess Alexandra Hospital (PAH) NHS Trust, 2018; Sommers et al., 2017). To inform continued development of feedback provision, it is important to understand the perceptions and attitudes of those clinicians that these mechanisms are intended to benefit (Eva et al., 2012). This study aimed to explore paramedics’ perceptions of the clinical performance feedback currently available and their attitudes towards the introduction of formal mechanisms for providing patient outcome feedback.

## Methods

A qualitative methodology using interpretative phenomenological analysis (IPA) of individual interview data was employed, enabling both descriptive and interpretive analysis (Smith et al., 2009). Phenomenological research focuses on how people experience specific issues, and IPA places emphasis on an individual’s experience in addition to themes found common to a number of participants (Smith et al., 2009). This separates it from many other qualitative methods, but it is argued that focusing on the insights of the individual allows the reader to evaluate the extent to which these perceptions are shared with others in a similar context (Smith et al., 2009, p.15). IPA has been criticised for its inherent subjectivity (Paley, 2017), and it must be noted that the lead author is a registered paramedic and that his own interest in feedback provision has undoubtedly had an influence during the interview process and in the analysis of accounts. McWilliam (2010) suggested that the subjective nature of the participant–researcher relationship should be endorsed if the methods, results and discussion are presented transparently and coherently. This study is presented with reference to the standards for reporting qualitative research (O’Brien et al., 2014).

IPA’s focus on the individual’s account confuses the concept of data saturation (Saunders et al., 2018), and the priority is to obtain sufficiently detailed narratives with subsequent thorough analysis (Smith et al., 2009). Paramedics were recruited from a single National Health Service (NHS) ambulance station in England. A large sample size is inappropriate for IPA (Smith et al., 2009), and it was felt that a suitable sample could be obtained from open invitation to this population, ensuring a range of perspectives were represented. Approximately 40 paramedics were based at this station, serving a mixed urban and semi-rural population. All paramedics at this single station received an e-mail invitation to participate, and posters promoting the study were displayed. Eight semi-structured, face to face interviews were audiotaped between November 2017 and April 2018. The duration of completed interviews ranged from 28 to 78 minutes. Interviews were conducted with five female and three male participants. They had had ambulance service careers ranging from 18 months to 32 years, with a median of 7 years.

Audio recordings were transcribed by a secure online service, and the lead author performed data analysis with reference to Smith et al. (2009), a process summarised by Rapley (2011). A single transcript is analysed initially, identifying themes and clustering them into connected areas. This process is then repeated for all transcripts before identifying any commonality of themes across narratives. Themes did not undergo respondent validation, as at this stage they are a product of researcher–participant interaction (Giorgi, 2010). Approval was obtained from the relevant university ethics committee and from the participating Trust. All paramedics provided informed, written consent to participate.

## Results

Descriptive data analysis identified themes related to both the formal and informal feedback processes currently in existence (Table 1) and to participants’ attitudes towards potential formal outcome feedback mechanisms (Table 2). Themes related to the anticipated effects and possible designs of an outcome feedback process were revealed.

**Table 1.** Themes relating to current clinical feedback provision.

Formal feedback provision	Informal feedback provision
Absent or inadequate Associated with complaints, commendations and debriefs Only available for high-impact incidents A lack of constructive critical appraisal Communication barriers of information overload and unidirectional delivery exist Absence of routine meaningful engagement may lead to feelings of clinical isolation	Requires clinician to initiate Geographical barriers Importance of timeliness of feedback Time constraints on staff in working hours Verbal medium Issues of patient confidentiality Insufficient depth of feedback provided

**Table 2.** Themes relating to formal provision of outcome feedback.

Anticipated benefits	Reassurance leading to confidence in practice Enhanced reflection on practice Facilitated development of clinical pattern recognition Increased awareness of wider healthcare system Improved engagement
Anticipated risks	Potential to damage confidence Inferences from feedback must be made with care Information overload
Which patient presentations?	Diagnostic uncertainty Incident closure Patients not conveyed to the ED Patients discharged from ED without treatment
Which mechanism?	Initiated by request from the individual clinician Electronically delivered OR Involving staff mediators Self-reflection OR Supported learning Must minimise resource demands of information capture and delivery to be sustainable

### Perceptions of current clinical feedback provision

#### Formal feedback provision

All paramedics perceived formal feedback as either absent or inadequate. Complaints, commendations and incident debriefs were the most frequently cited sources, though the extent to which these constituted useful appraisal of clinical performance was questioned. Debriefing was reported to exist only for highly traumatic incidents, and their timing soon after an incident led participants to perceive that emotional considerations and lack of outcome information reduced the presence of constructive appraisal:

It’s almost wishy-washy-like [the debrief]. I don’t think anyone wants to offend their colleagues. (Paramedic G)

I don’t think you can fully evaluate that [did we do everything that we could do, pre-hospital?] unless you have some outcome, have some feedback from the hospital as to what has actually happened to this person. (Paramedic D)

Clinical updates delivered online or on training days were identified as a form of feedback, as they were perceived to originate from audit and serious incident investigations. The frequency and volume of online and paper-based communications appeared to limit engagement. Furthermore, two paramedics felt that information was delivered in a unidirectional top-down manner, with no subsequent engagement with the intricacies of implementation in practice:

[clinical updates are] instructions, and it’s expected that you follow them. But there isn’t any follow-up to see how that’s gone, or to see how it fits with real life, you know? (Paramedic B)

A lack of meaningful appraisal and formal engagement with daily practice appeared to result in some participants describing a feeling of clinical isolation:

You only know you’re on the right track because you’re not getting complaints, and that’s the wrong way to do it. (Paramedic B)

#### Informal feedback provision

In the absence of formal processes, participants repeatedly confirmed the use of informal feedback mechanisms to discover patient outcomes. The barriers and limitations to this process were highlighted:

Even, sometimes, if you do get to the hospital, it’s difficult because you don’t know who is looking after them. If the patient has already been moved out of A&E, and gone, you can’t track them down … I want to know what the doctor’s examination was, what they found and what their plan is for treating them. I think, sometimes, the nurses can’t always give me that kind of depth – unless they’re going to let me look at the notes and actually read through what happened. (Paramedic A)

### Attitudes towards formal provision of outcome feedback

#### Anticipated benefits and risks

All of the paramedics felt that outcome feedback would benefit confidence in practice, enhance reflection and improve their diagnostic skills by enabling patterns of clinical presentation to be learnt. Some paramedics also anticipated development of decision-making skills from an improved understanding of the wider patient experience:

The practical reality is that we don’t deal with the longer-term concerns of our patients. If you want paramedics to be more confident in their ability to treat patients as an isolated care episode, then understanding the way that they’re going to be treated in the longer term is a massive part of how you would be able to safely make that decision. (Paramedic C)

Another benefit highlighted was enhanced work engagement enabled by incident closure:

[Outcome feedback] would also make you become a bit more attached to the job, because you know what’s happened from start to finish with that patient, rather than now we might take them to hospital and that’s it, job done, next job. I guess it would give you a bit of closure on some jobs, particularly ones that have touched you in an emotional way. (Paramedic E)

Participants recognised that developmental benefits could be gained from feedback that challenged practice undertaken, but highlighted that this had potential to undermine confidence. Many participants were also mindful that feedback is subject to interpretation and any inferences drawn from it must be made with care and based upon sufficient information.

#### Which patients and what mechanism?

Feedback was most commonly desired for patients with diagnostic uncertainty. Additionally, all participants demonstrated motivations of curiosity and compassion underlying a desire for outcome information:

I went to the stroke ward to ask what had happened, because it was quite a severe one and it really stuck in my mind; I wanted to know what had happened. (Paramedic E)

Receiving feedback on patients who had seemingly not benefitted from transport to the ED and from community care providers was anticipated to benefit understanding of secondary and primary care processes respectively. Participants highlighted that this understanding might enable the paramedic role to develop, performing new functions to benefit the cohesion of the whole healthcare system. Some felt that outcome feedback would ideally be available for all patients encountered, though others were wary of information overload.

Unanimously, paramedics suggested that their request would initiate the feedback process. Information accessed from electronic patient record systems, in a similar way that general practitioners (GPs) receive hospital discharge summaries, was the most commonly suggested method of delivery. Some also suggested that peer mediators could source and deliver information:

I think from certain jobs, especially having the iPads, you know, if you could flag it, a PCR, and say on it, maybe, a tick box to say, ‘I want feedback from this particular job’, then it wouldn’t be every job, then, and you would have specific incidents that you were asking for feedback for. Someone would then go off and find the information, and come back to you on it specifically. (Paramedic B)

The GPs already use that, a system where they get the patient details from an A&E visit and they can see, from start to finish, their treatment. Adopting something like that that we could have access to on our iPads that we’ve already got would be great. (Paramedic H)

Attitudes were polarised among paramedics concerning whether feedback should be provided for self-reflection or should involve supervisors for supported provision:

Yes, you definitely need a process there to support you. There wouldn’t be any point in just giving feedback and then making a load more questions for yourself to answer. You would need someone to go to, to try and discuss it with, like a form of debrief I suppose. Yes, just to make sure some sort of learning has occurred from it. (Paramedic E)

I don’t know. I’m not so keen on that idea [feedback delivered with supervision], I don’t know why . . . Some of them, maybe, don’t need discussion as such – it’s more, just literally, finding out that bit of information. Once you’ve got that information, you can maybe look that up on your own. (Paramedic A)

This may depend upon individual learning preferences but the potentially damaging effects of negative feedback were perceived as necessitating careful consideration of how it is delivered and perhaps a level of external monitoring.

Finally, many paramedics stressed that to be sustainable, a feedback process must acquire and deliver information efficiently, minimising resource demands.

### Clinician and culture

**Figure 1:**
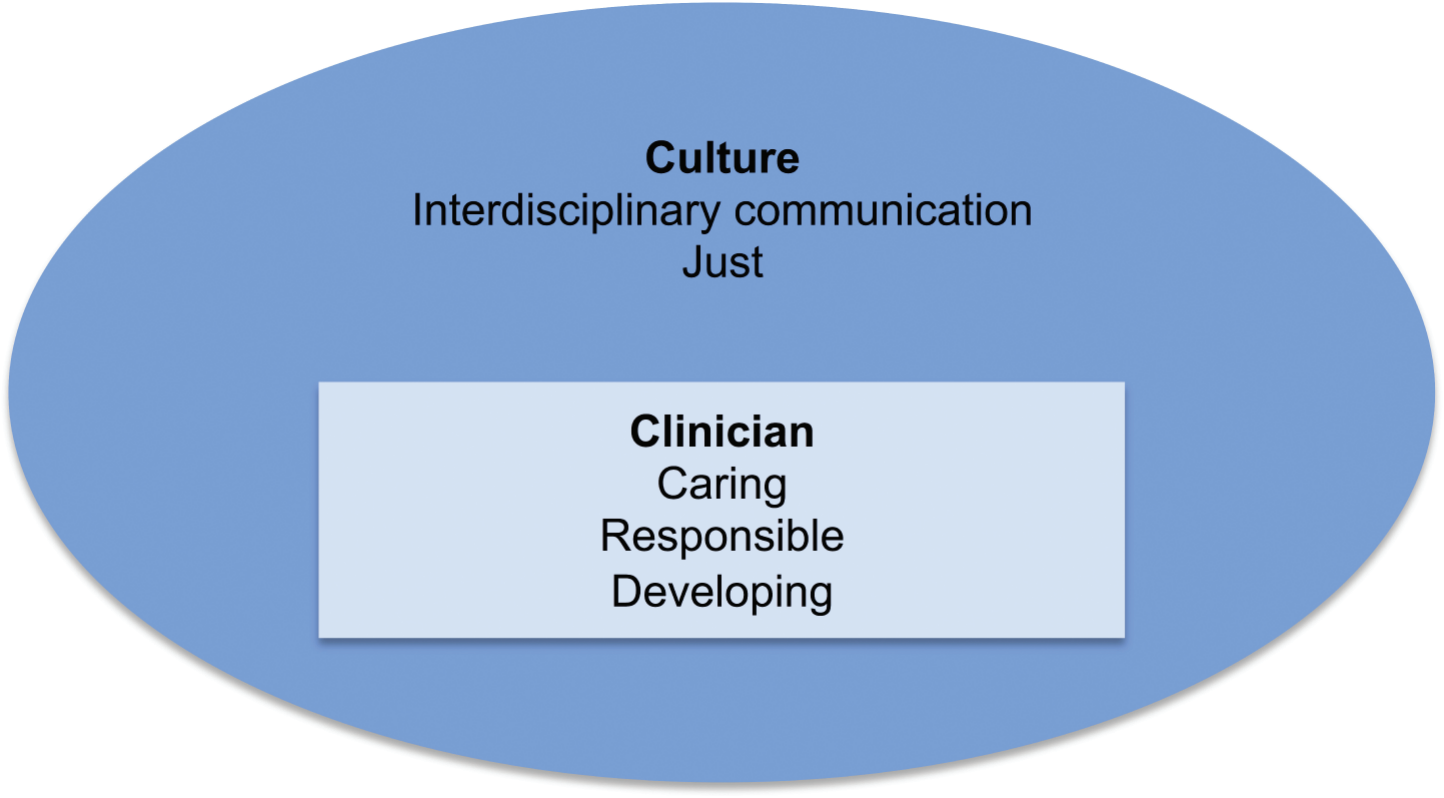
Figure 1. Interpretative themes concerning clinician and culture.

Interpretative data analysis revealed qualities and desires demonstrated by paramedics during discussion of feedback concerning both their individual practice and their working environment or culture (Figure 1).

It was apparent during interviews that there was often substantial emotional involvement in incidents, and all of the paramedics demonstrated significant professional responsibility for the well-being of their patients:

She [a paramedic colleague] phoned me at 5 in the morning having finished at 2 o’clock in the morning because she got home and she was just worried about this patient she’d left at home, really worried, couldn’t sleep. (Paramedic E)

Participants repeatedly expressed a strong commitment both to individual clinical development and to the development of the paramedic professional role. One paramedic highlighted the discrepancy between feedback received as a student and that available following qualification, remarking that the need to develop clinically does not disappear following registration. Another expressed an almost envious perception that other professions had access to information concerning outcomes and utilised this effectively to develop:

You see nurses with an amazing amount of information and diagnostic capability, well beyond their training. But they haven’t been taught; they’ve acquired it, because they’re seeing these patients on a day-to-day basis, and they’re knowing what’s happening to them. They’re knowing the outcomes. (Paramedic C)

Participants expressed a need for effective communication to exist between health professionals of all disciplines, enabling mutual learning and understanding. However, they perceived that this must be accompanied by a culture where clinicians are encouraged and supported to learn from their mistakes without inappropriate blame attribution; a ‘just culture’:

There wasn’t anything positive. There was no positive outcome from it [the debrief]. Everything was negative. It was all blame. (Paramedic G)

## Discussion

In line with previous studies, these paramedics perceived a dearth of formally enabled feedback to ambulance clinicians (Cash et al., 2017; O’Hara et al., 2015). Additionally, feedback provided was perceived as reserved for exceptional incidents and lacking sufficient constructive appraisal to benefit development, a finding shared by Morrison et al. (2017). Similarly, Couper and Perkins (2013) suggested that debriefing conducted a longer period of time after the event incorporating feedback of outcomes or performance measures may be more effective in improving practice than the traditional ‘hot debrief’ immediately following an incident.

This study suggests that addressing the clinical isolation expressed by some paramedics (Hörberg et al., 2017; O’Hara et al., 2015) may demand new processes, as participants highlighted perceptions of information overload and instructional inflexibility associated with existing communication methods. All participants confirmed the use of informal channels for obtaining outcome information. Yet Ivers et al. (2012) advocated that effective feedback is delivered repeatedly, not solely in conversation and with a resultant action plan, seemingly necessitating a more structured formal approach. Perhaps formal feedback offers an effective means of communication with the ambulance clinical workforce, permitting meaningful appraisal of day-to-day performance and reducing impressions of clinical practice isolation.

Paramedics anticipated that outcome feedback would benefit well-being and work engagement in addition to individual professional development. They demonstrated qualities of compassion and responsibility, together with a desire to develop when discussing feedback. Current feedback systems in the UK recognise this benefit (PAH NHS Trust, 2018; Sommers et al., 2017), and it seems possible therefore that feedback provision may have some potential to reduce the high levels of work-related stress, absenteeism and retention difficulties seen in the ambulance service workforce currently (National Audit Office, 2017).

A greater awareness of other organisational processes and capabilities was anticipated to improve future decision-making. If this process is bi-directional then inter-disciplinary understanding will profit. This has been demonstrated by studies of feedback in specific patient care pathways such as those for acute myocardial infarction (AMI) and cerebrovascular accident (CVA) (Choi et al., 2014; Daudelin et al., 2010; Scholz et al., 2008; Siriwardena et al., 2014). Greater understanding of previously distinct elements of the patient journey, of the importance of each role and of the various problems encountered in practice improved the cohesion of the condition-specific pathway. Similarly, Sheffield et al. (2016) concluded that inter-professional collaboration improved appropriate community referral rates by paramedics. Thus this benefit applies for feedback supplied by both secondary and primary care services.

Improved diagnostic and decision-making skills were benefits anticipated to enable safer autonomous practice and more effective navigation of the healthcare system. In addition to individual development, participants were motivated to develop the paramedic role to facilitate accessing the most effective journey for the patient. Integration of emergency and urgent care providers, with a paramedic navigator role, is a strategic vision for the development of the ambulance service in the UK (Association of Ambulance Chief Executives (AACE), 2015). Formal outcome feedback provision might be a facilitator to achieving these goals. Yet, this study suggests that if open and honest interdisciplinary communication is to exist, it must be accompanied by a supportive and just culture. If there is to be greater organisational involvement with a paramedic’s day-to-day performance to benefit effectiveness and continued engagement, then openness must be accompanied by fairness and facilitation of development (Eva et al., 2012; NHS Improvement, 2018).

Paramedics predominantly described electronic access to outcome information. Unfortunately, the development and compatibility of electronic record systems currently does not support such a system (Porter et al., 2018) and there are information governance concerns associated with sharing patient data in this manner (Eburn, 2017). However, this level of electronic interoperability across healthcare providers remains the NHS goal (Wachter, 2016), and one feedback system currently in operation has recently had a system of assumed patient consent to data sharing approved (PAH NHS Trust, 2018). It seems likely, therefore, that the creation of electronic outcome feedback mechanisms operating between NHS providers may be facilitated in the future.

Initiation of a feedback process by the paramedic may limit its effectiveness, as opportunities for learning may not always be apparent to the clinician (Hodell et al., 2016). Yet feedback on all patients encountered (Pollard & Black, 2015) was felt by some to be excessive and would allow the clinician to decide which incidents they engaged with anyway. Feedback provision initiated by the provider (Brichko et al., 2016) would require resources to identify the requirement for feedback, and delivery would need to be carefully considered to avoid negative perceptions. A resource-demanding process of this type would realistically predominantly be used to highlight corrections to practice rather than to reinforce practice undertaken. Yet literature suggests that balanced feedback is required to maintain engagement and that effective feedback delivery must come from a clear position of beneficent intent (Eva et al., 2012; Hodell et al., 2016).

There was a difference of opinions on whether feedback provision should be mediated or for self-reflection. Monitored or supported feedback mechanisms will necessitate greater resources; a barrier identified for sustainable delivery. Additionally, paramedics may be reluctant to have perceived errors recognised formally and therefore engagement with a supervised mechanism may be reduced (Eva et al., 2012; Morrison et al., 2017). Yet the effectiveness of self-assessment without further external guidance has been questioned (Archer, 2010; Sargeant et al., 2010). Feedback delivered by credible peers is advocated as a preferable strategy (Ivers et al., 2012). Thus, results from this study suggest that while electronic feedback provision, accessible for self-assessment, may be most sustainable, care must be taken to ensure that sufficient information is provided for the clinician to understand how the patient arrived at the outcome disclosed. Furthermore, access to an appropriately experienced colleague for the purposes of support and assisting development should be formally enabled and clearly signposted (Archer, 2010).

## Limitations

This study is limited as data was taken from a single ambulance station employing a single method of collection. Additionally, sampling by invitation means that those who have little interest in feedback are unlikely to participate. However, the results of this study are presented for the reader, with some shared experience, to gauge the transferability of the themes identified, and many of them are supported by international literature review.

## Conclusions

Effective and just provision of outcome feedback was anticipated to reduce isolation of the paramedic role; isolation from learning the conclusions of events paramedics have been involved in, from their own professional organisation and from other health and social care providers. This was expected to enhance both the clinical development of the individual and the integration of the paramedic profession within the wider healthcare system. In addition, feedback was expected to benefit individual well-being and work engagement in a profession currently experiencing significant work-related stress and retention difficulties. Though electronic delivery of feedback for self-reflection will reduce resource demands, inherent risks were perceived, by some, to necessitate external sources of supervision and support.

This study has revealed some paramedics’ attitudes towards formal delivery of outcome feedback. Further research is required to evaluate the benefits and risks associated with outcome feedback provision in practice.

## Author contributions

PEW was responsible for the conception, design, data acquisition, analysis and interpretation of the article, as well as drafting and amending it. FM and CM supervised design of the study, data analysis and interpretation and conducted a critical review of the submission. PEW acts as the guarantor for this article.

## Conflict of interest

None declared.

## Ethics

University of Surrey, Faculty of Health and Medical Sciences Ethics Committee. Approval to conduct the study was also attained from the South East Coast Ambulance Service NHS Foundation Trust Research and Development Group.

## Funding

This study was pursued to complete PEW’s MSc dissertation module. The module fee was partially funded by PEW’s employer, South East Coast Ambulance Service NHS Foundation Trust, though that funding was not used to conduct the study.
